# Outcomes of robotic anatomic lung resection after neoadjuvant therapy for non-small cell lung cancer

**DOI:** 10.3389/fsurg.2026.1765234

**Published:** 2026-05-20

**Authors:** Camille Yongue, Ammar Asban, Ashley McCormack, Caroline A. Snyder, Isabella Ferraro, Nikolaos Pachos, Michael Zervos, Robert Cerfolio

**Affiliations:** 1 Department of Cardiothoracic Surgery, NYU Langone Health, New York City, NY, United States; 2 Department of Thoracic Surgery, RWJ Barnabas, West Orange, NJ, United States; 3 NYU School of Medicine, New York City, NY, United States

**Keywords:** lobectomy, neoadjuvant, NSCLC, pneumonectomy, robotic surgery, thoracic surgery

## Abstract

**Background:**

Previous studies report a 20% conversion to open thoracotomy and 25% major morbidity rate for minimally invasive thoracic surgery following neoadjuvant chemotherapy or immunotherapy.

**Methods:**

This retrospective review includes a consecutive (non-selected) series of patients from two surgeons who underwent robotic resection after neoadjuvant therapy for non-small cell lung cancer.

**Results:**

From January 2018 to October 2024, 150 patients (51% male) underwent surgery following systemic therapy. The median age was 67 years. Preoperatively, 92% received chemotherapy, 65% immunotherapy, and 27% radiation. Median time from therapy to surgery was 6 weeks. The most common tumor type was stage IIIA adenocarcinoma (25%). Median operative time was 152 min, and median blood loss was 20 mL. There were no unplanned conversions to open thoracotomy or from lobectomy to pneumonectomy. Median length of stay was 1 day; 28% had chest tube removed on the day of surgery. Twenty-one patients experienced Clavien-Dindo grade III complications (primarily atrial fibrillation and effusion). There were no 30-day mortalities and two 90-day mortalities. Median follow-up was 19 months, with a median postoperative survival of 513 days.

**Conclusion:**

Robotic lobectomy and pneumonectomy can be safely performed after neoadjuvant therapy, with conversion rates <1% and minimal 30- and 90-day mortality. Key technical factors include intra-pericardial control of the pulmonary artery, division of the lobar airway prior to pulmonary artery dissection, and performing surgery within 6 weeks of completing neoadjuvant therapy.

## Introduction

Neoadjuvant therapy has increasingly been used for stage IB-IIIA NSCLC to reduce tumor size, eliminate circulating micrometastases, and decrease recurrence rates (1, 2).

Neoadjuvant therapy may include chemotherapy, immunotherapy, and/or radiation therapy. The optimal timing of surgery is after the patient has achieved maximal therapeutic benefit, as determined by repeat CT and integrated PET imaging or other objective assessments such as circulating tumor DNA or MRI. According to Guo et al., the optimal interval for surgery is 2–6 weeks after the final dose of neoadjuvant therapy, once maximal response has been achieved (3).

Surgical resection after neoadjuvant therapy is more technically challenging due to inflammatory changes in the hilar lymph nodes and frequent loss of tissue planes. Minimally invasive approaches, such as robotic resection, have increasingly been utilized and have been shown to be safe and feasible (4, 5)^,^.

We present a single center, two surgeon experience of 150 consecutive patients who underwent robotic lobectomy or pneumonectomy after neoadjuvant therapy between 2018 and 2025, which represents, to our knowledge, the largest series to date.

## Methods

This is a retrospective review of a prospectively maintained database including a consecutive (non-selected) series of patients who underwent robotic lobectomy or pneumonectomy after neo-adjuvant therapy for NSCLC from 1/2018 to 12/31/2024. Patients who received neo-adjuvant chemotherapy, immunotherapy, radiation or any combination thereof were included in the analysis.

Data collected included demographics, preoperative oncologic variables, operative details, pathology, and postoperative outcomes including complications. The institutional review board at NYU Langone approved this study.

Primary outcomes were length of stay, R0 resection, conversion rate, readmission, morbidity and mortality. Secondary outcomes included operative time, lymph node yield, estimated blood loss, and chest tube duration. Morbidity was defined using the Clavien-Dindo classification (6).

All patients underwent standard robotic positioning and port placement. A complete mediastinal lymph node dissection was performed in all cases; including levels 7, 8, 9, 10, 11, and 12, and levels 2 and 4 (right) or 5 and 6 (left). In neoadjuvant cases, a low threshold was maintained for obtaining proximal and distal pulmonary artery control in the setting of a hostile hilum. A combination of robotic and bedside stapling was used to divide structures.

All procedures were performed using the Da Vinci XI robotic system (Intuitive Surgical, Inc). Continuous variables are reported as median with interquartile range. Statistical analyses were performed using Microsoft Excel and Stata/SE 18.0 (StataCorp LLC), with a two-sided significance level of *P* < .05. IRB approval number: i24-00978 (September 20, 2024).

### Propensity score matching

To provide a control cohort, a propensity score–matched analysis was performed between patients undergoing robotic pulmonary resection after neoadjuvant therapy and a contemporaneous cohort undergoing robotic resection for primary lung cancer, by the same two surgeons. Propensity scores were estimated using multivariable logistic regression based on pre-treatment covariates, including age, sex, body mass index, ECOG performance status, FEV1 (% predicted), histology, clinical T stage, nodal status, and year of surgery. Matching was performed using nearest-neighbor matching without replacement in a 1:2 ratio, with a caliper of 0.2 standard deviations of the logit of the propensity score. Covariate balance was assessed using standardized mean differences, with values <0.10 indicating adequate balance. Within the matched cohort, continuous variables were compared using the Mann–Whitney U test and categorical variables using chi-square or Fisher's exact tests, with a two-sided *p*-value <0.05 considered statistically significant. Analyses were performed using Python (version 3.9).

## Results

From January 1, 2018 to December 31, 2024, 150 patients underwent resection of NSCLC after systemic therapy. Table 1 shows patient demographics including pre-operative ECOG performance status, pre-operative pulmonary function, pre-operative smoking status and pre-existing comorbidities including those requiring pre-operative anticoagulation. Systemic treatments for NSCLC included pre-operative chemotherapy in 138 patients (92%), immunotherapy in 97 patients (65%) and radiation therapy in 41 (27%). Median time from neoadjuvant therapy to surgery was 6 weeks. The most common chemotherapy regimen was carboplatin or cisplatin combined with pemetrexed or paclitaxel, a platinum-based regimen combined with etoposide was also used in some cases. The most common immunotherapy/targeted therapy regimens were pembrolizumab, osimertinib, erlotinib, nivolumab and atezolizumab.

**Table 1:** Pre-operative characteristics of patients who underwent lung resection after neo-adjuvant therapy.

Characteristic	Neo-adjuvant (Chemo/Immuno/Radiotherapy) prior to surgery (*N* = 150)
Age- yr Median [range]	67 [38, 88]
Male sex – no. (%)	76 (51%)
Race – no. (%)	
White	112 (75%)
Black	7 (5%)
Other	31 (21%)
BMI – no. Avg [Std Dev]	26 [+/−5]
ECOG (%)	
0	43%
1	48%
2	6%
>2	0
FEV1 - % Mean [Std Dev]	89% [+/−21]
DLCO - % Avg [Std Dev]	76% [+/−18]
Smoking status at the time of surgery– no. (%)	
Never smoker	45 (30%)
Former smoker	93 (62%)
Current smoker	11 (7%)
Pack year history – years Median [range]	20 [0,100]
HTN – no. (%)	75 (50%)
DM – no. (%)	17 (11%)
CAD – no. (%)	23 (15%)
CKD – no. (%)	12 (8%)
Lung disease	31 (21%)
COPD	21 (14%)
Emphysema	29 (19%)
Pulm HTN	0 (0%)
Pre-operative anti-coagulation – no. (%)	
Plavix	6 (4%)
Direct oral anticoagulant (DOAC)	15 (10%)
Warfarin	1 (1%)
Other	2 (1%)

 Table 2 shows pre-operative tumor type and stage. The most common tumor type was adenocarcinoma (65%) and stage was stage IIIa (27%). Median pre-operative tumor size was 3.3 cm (range 0.65–13.1 cm).

**Table 2:** Pre-operative treatment and stage.

Characteristic	Neo-adjuvant (Chemo/Immuno/Radiotherapy) prior to surgery (*N* = 150)
Tumor type – no. (%)	
Adenocarcinoma	97 (65%)
Squamous cell carcinoma	29 (19%)
Other NSCLC	8 (5%)
Pre-operative final pathologic stage – NSCLC 8th edition – no. (%)	
IA1	0 (0%)
IA2	3 (2%)
IA3	1 (1%)
IB	7 (5%)
IIA	4 (3%)
IIB	16 (11%)
IIIA	40 (27%)
IIIB	23 (15%)
IV A/B	54 (36%)
Pre-operative size – cm Median [range]	3.3 cm, (0.65–13.1 cm)
Time to surgery (median)	6 weeks (0, 180)

 Table 3 shows intra-operative outcomes. The most common type of resection was lobectomy (88%) followed by bi-lobectomy and pneumonectomy (6%). There were two planned robot assisted thoracotomies, one for extensive radiation changes producing anastomotic tension at the construction of the right main stem to the bronchus intermedius and the other to complete chest wall resection. Median EBL was 20cc and there was 1 intra-operative transfusion. Twenty-four patients required intra-pericardial PA control, and 14 patients underwent PA plasty to achieve an R0 resection. Types of PA plasty included partial PA resection as well as circumferential PA excision with an end to end anastomosis.

**Table 3:** Intra- operative outcomes.

Characteristic	Neo-adjuvant (Chemo/Immuno/Radiotherapy) prior to surgery (*N* = 150)
Planned robotic approach – no. (%)	147 (98%)
Planned robot assisted approach – no. (%)	2 (1%)
Lobectomy – no. (%)	132 (88%)
Bilobectomy – no. (%)	9 (6%)
Pneumonectomy – no. (%)	9 (6%)
Sleeve – no. (%)	3 (2%)
Operation length – minutes Median [range]	155 min (50, 455)
Intra-operative transfusion – no. (%)	1 (1%)
Intra-operative complication – no. (%)	0 (0)
Estimated blood loss (EBL) – mL Median [range]	20 mL [5,650]
Intra operative central PA control	24
Intra operative PA plasty	12

 Table 4 shows short term post operative outcomes. 145 of 150 patients did not require and intensive care unit (ICU) stay. Median length of stay was one day. Median day of chest tube removal was post-operative day 1 (0,10), forty patients had their chest tube removed the day of surgery. There were 15 patients who had post operative atrial fibrillation requiring pharmacologic intervention (Clavien Dindo II). There were 6 patients who required post operative tube thoracostomy for removal of air or fluid (Clavien Dindo III). Air leak requiring a chest tube for >1 day was not classified as a complication but rather within the expected post operative course for a complex neo-adjuvant resection. There were 31 patients discharged home with a chest tube (21%). There were no instances of post operative pneumonia, thromboembolic events or return to the operating room for re-intervention. There were 2 mortalities at 90 days.

**Table 4:** Post operative outcomes.

Characteristic	Neo-adjuvant (Chemo/Immuno/Radiotherapy) Prior to Surgery (*N* = 150)
Intensive Care Unit (ICU) Stay Post Op, no. (%)	5 (3%)
Length of Stay (LOS) – days Median [Range]	1 day [1,41]
Day Chest Tube Removed– days Median [Range]	1 day [0,10]
Patients Discharged with Chest Tube – no. (%)	31 (21%)
Post Operative Morbidity (Clavien Dindo II) – no. (%)	15 (10%)
Clavien Dindo III – no. (%)	6 (4%)
R0 Resection – no. (%)	143 (95%)
Re-Admission – no. (%)	17 (11)
30 day Mortality – no. (%)	0 (0%)
90 day Mortality – no. (%)	2 (1%)

Table 5 summarizes final tumor pathology. The median number of N2 stations resected was 19 (5 stations) and median number of N1 lymph nodes removed was 7 (3 stations). Post operatively, 24 patients were found to have a complete response on final pathology (16%), 21 patients were restaged to IA1 and 14 were restaged to IA2 (see Table 5 for complete breakdown of the yp staging). An R0 resection was achieved in 95% of patients.

**Table 5:** Final pathologic response.

Characteristic	Neo-adjuvant (Chemo/Immuno/Radiotherapy) prior to surgery (*N* = 150)
N1 nodes on final pathology - number Median [range]	7 [0,35]
N2 nodes on final pathology – number Median [range]	19 [1,59]
Post-operative final pathologic stage (yp)– NSCLC 8th edition – no. (%)	
Stage 0 (Complete response)	24 (16%)
IA1	21 (14%)
IA2	14 (9%)
IA3	5 (3%)
IB	7 (5%)
IIA	3 (2%)
IIB	13 (8%)
IIIA	32 (21%)
IIIB	8 (5%)
IV A	1 (1%)
IV B	17 (11%)

**Figure 1:**
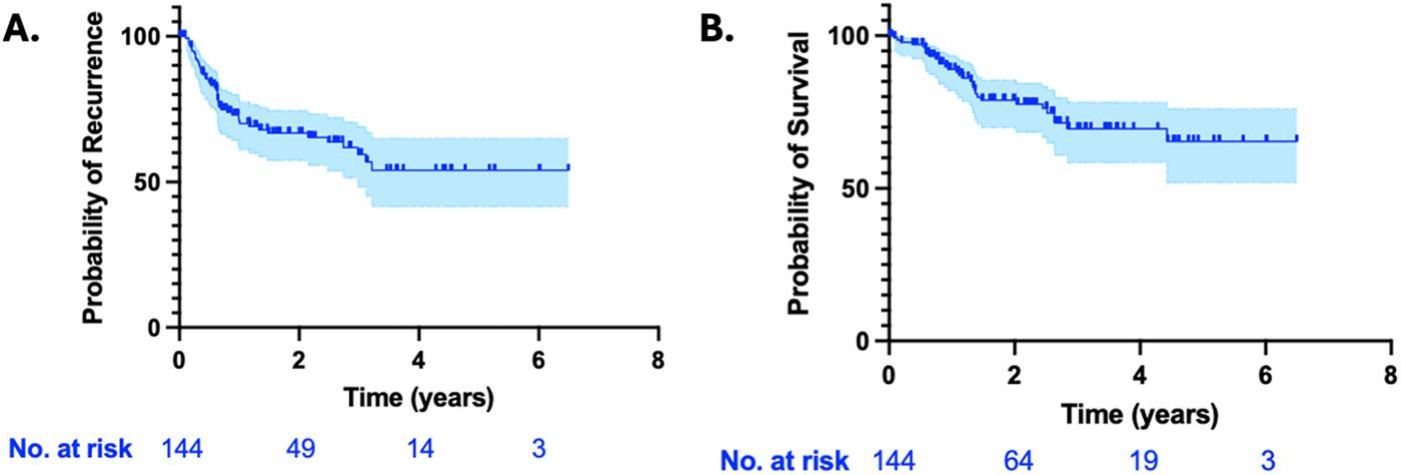
Kaplan–Meier analysis of recurrence **(A)** and overall survival **(B)** calculated from the date of surgery in the entire cohort of patients with follow-up data (*n* = 144).

 Table 6 shows mid to long term outcomes. Follow up was complete in 96% of patients with a median of 19 months (min 0, max 77), Missing data was excluded from analysis. 113 patients were alive at the time of last follow up. 109 (73%) patients received further treatment post operatively; 57 (38%) received adjuvant chemotherapy, 91 (61%) received adjuvant immunotherapy and 48 (32%) received adjuvant radiation. Figure 1 shows the Kaplan–Meier analysis of recurrent and overall survival calculated from date of surgery.

**Table 6:** Mid to long term survival.

Characteristic	Neo-adjuvant (Chemo/Immuno/Radiotherapy) prior to surgery (*N* = 150)	
Long term follow up	144 (96)%	
Follow up duration (months)- median (range)	19 months (0–77)	
Recurrence – *n* (%)	48	33%
Time from surgery to recurrence (months) –	7 (2–38)	
Status at last follow-up – *n* (%)		
Alive	113	78%
Deceased	31	22%
Time from surgery to death (months)	13 (1–72)	
Adjuvant therapy- n(%)	109	73%
-Chemotherapy	57	38%
-Immunotherapy	91	61%
-Radiation	48	32%

 Table 7 demonstrates outcomes compared with a historically matched cohort from our institution who underwent primary robotic lung resection for early stage NSCLC.

**Table 7:** Perioperative outcomes in propensity score–matched cohort.

Variable	Neoadjuvant (*n* = 68)	Control (*n* = 131)	*p*-value
Operative details			
Operative time, min	148 [105–192]	122 [104–155]	0.073
Length of stay, days	1 [1–2]	1 [1–2]	0.856
Conversion to open thoracotomy	1 (1.5%)	0 (0.0%)	0.342
ICU admission	1 (1.5%)	2 (1.5%)	0.594
Postoperative complications			
Any complication	12 (17.6%)	21 (16.0%)	0.841
Pneumonia	3 (4.4%)	0 (0.0%)	0.039
Atrial fibrillation	6 (8.8%)	4 (3.1%)	0.094
Myocardial infarction	0 (0.0%)	0 (0.0%)	1.000
Prolonged air leak (discharged with chest tube)	10 (14.7%)	19 (14.5%)	1.000
30-day outcomes			
Readmission	8 (11.8%)	15 (11.5%)	1.000
Mortality	0 (0.0%)	0 (0.0%)	1.000

Data are median [interquartile range] or *n* (%). *p*-values: Mann–Whitney U test (continuous) and Fisher's exact test (categorical).

Propensity score matching yielded 68 patients in the neoadjuvant group matched to 131 patients undergoing upfront surgery. Baseline characteristics were well balanced between groups, with acceptable standardized mean differences. In the matched cohort, operative time was longer in patients undergoing resection after neoadjuvant therapy, though this did not reach statistical significance [median 148 (IQR 105–192) vs. 122 (IQR 104–155) minutes; *p* = 0.073]. Postoperative pneumonia was more frequent in the neoadjuvant group (4.4% vs. 0.0%; *p* = 0.039). All other perioperative outcomes were similar between groups, including length of stay [median 1 (IQR 1–2) vs. 1 (IQR 1–2) days; *p* = 0.856], overall complications (17.6% vs. 16.0%; *p* = 0.841), atrial fibrillation (8.8% vs. 3.1%; *p* = 0.094), prolonged air leak requiring discharge with a chest tube (14.7% vs. 14.5%; *p* = 1.000), and 30-day readmission (11.8% vs. 11.5%; *p* = 1.000). There were no 30-day deaths in either group.

## Discussion

This series demonstrates the safety and efficacy of robotic pulmonary resection following neoadjuvant therapy for NSCLC, even in stage IV disease, with lower morbidity and mortality than reported in previous studies.

Previous studies report a 20% conversion to open and 25% major morbidity rates for minimally invasive thoracic surgery after neo-adjuvant chemo- and/or immunotherapy and radiotherapy (7, 8). More recent studies have shown improvement in perioperative morbidity and mortality but have a small sample size and longer length of stay (9). For example, a recent retrospective study of patients who underwent robotic lobectomy for NSCLC after neoadjuvant therapy was 44 patients with average LOS 6.5 days (2022) (10).

The prior largest study of robotic surgery after neo-adjuvant therapy was conducted at a specialty hospital in China at Central South University Hospital, Hunan on 142 patients. Conversion to thoracotomy was 7.5% and average LOS was 6 days (11).

At our institution (a high volume tertiary care center), nearly all lung resections are performed robotically with an expected length of stay of 1 day (12). Our results have been previously published-all patients are enrolled in the enhanced recovery after surgery (ERAS™) pathway that includes pre-operative analgesia, extubation in the operating room, ambulation within two hours post op and early chest tube removal (13). Published outcomes for elective robotic thoracic surgery (in a non-neoadjuvant setting) from this institution are: 97% of segmentectomies and 68% of lobectomies discharged by post operative day one, and 12% discharged home with a chest tube with 0.3% morbidity and mortality. This neoadjuvant cohort has similar if not better post operative outcomes.

Patients in this cohort were scheduled within 6 weeks or less of the completion of neoadjuvant therapy The two surgeons queried in this retrospective review each perform an average of ∼300 cases a year, and approximately two-thirds of their cases are anatomic lung resections. They both frequently perform robotic neo-adjuvant resections (14). This specific experience and the robust pathways in use at this hospital may make these results difficult to generalize.

In addition to specific intra-operative techniques that help accomplish these outcomes. These techniques are shown in the attached Supplementary Videos vignettes. First, there is a low threshold to obtain proximal and distal pulmonary artery (PA) control (including distal control on the inferior pulmonary vein) which allows for the PA to become depressurized and more safely dissected. Second, division of the lobar airway is performed prior to pulmonary artery dissection if the surgical planes are adhered in order to safely visualize each structure. The lobar airway is then sewn closed or stapled more proximally. Third, there is a low threshold to call in a second surgeon experienced with robotic anatomic lung resections, and there is a robust culture of teamwork.

Limitations of this study include single center experience, heterogeneity of the pre-operative therapy received, and possible patient selection bias as this is a center of referral for advanced lung cancer. All patients are presented at a multi-disciplinary tumor board and selected for resection based off of expert consensus. The group of patients selected for resection likely has a greater functional status and higher PFTs than the broader population of patients with advanced NSCLC. There is also a skew towards an increase in the number of neoadjuvant cases performed per year at this institution from 2018 to 2025.

Further study is needed to determine optimal surgical timing, the extent of resection that is oncologically appropriate and whether there are pre-operative features that can predict a complete response.

## Central picture legend

Our Scalable Process for Performing Robotic Resection after Neo-Adjuvant Therapy for NSCLC

## Central message

Patients with advanced NSCLC who receive neo-adjuvant treatment can undergo robotic resection with safety and outcomes equivalent to those with early-stage untreated non-small cell lung cancer (NSCLC).

## Perspective statement

Patients with advanced NSCLC are increasingly receiving neoadjuvant chemoimmunotherapy prior to surgery. Previous reports suggest high conversion rates from a robotic approach to open thoracotomy and morbidity rates of approximately 25%. We report a series of 150 patients- the largest published to date- who achieved R0 resection with lower morbidity and mortality using a robotic approach, with a conversion rate of 1%.

## Data Availability

The raw data supporting the conclusions of this article will be made available by the authors, without undue reservation.
