# Supplementary material for: Outcomes of robotic anatomic lung resection after neoadjuvant therapy for non-small cell lung cancer
Source: Front Surg. 2026 May 20;13:1765234. doi: 10.3389/fsurg.2026.1765234 (PMC13231731; doi:10.3389/fsurg.2026.1765234)
Supplement: Supplementary file 1 [file Table1.docx]

Supplemental Material

| Supplementary Table S1. Baseline Characteristics After Propensity Score Matching | | | | |
| --- | --- | --- | --- | --- |
| Variable | **Neoadjuvant (n = 68)** | **Control (n = 131)** | **p-value** | **SMD** |
| Age, years | 69.0 [61.8–74.2] | 66.0 [59.0–73.5] | 0.456 | 0.064 |
| Male sex | 30 (44.1%) | 63 (48.1%) | 0.654 | 0.080 |
| BMI, kg/m² | 25.9 [22.6–30.1] | 25.9 [22.4–28.6] | 0.918 | 0.025 |
| ECOG performance status | 0 [0–0] | 0 [0–0] | 0.346 | 0.105 |
| FEV₁, % predicted | 89.0 [76.0–104.0] | 87.0 [74.0–101.0] | 0.557 | 0.130 |
| DLCO, % predicted | 80.0 [66.2–95.2] | 78.0 [64.0–88.0] | 0.633 | — |
| Histology |  |  |  |  |
| Adenocarcinoma | 48 (70.6%) | 100 (76.3%) | 0.396 | 0.130 |
| Squamous cell carcinoma | 11 (16.2%) | 18 (13.7%) | 0.675 | 0.068 |
| Other / not specified | 9 (13.2%) | 13 (9.9%) | — | — |
| Resection type |  |  |  |  |
| Lobectomy | 63 (92.6%) | 123 (93.9%) | 0.767 | 0.050 |
| Pneumonectomy | 1 (1.5%) | 6 (4.6%) | 0.426 | 0.182 |
| Bilobectomy / sleeve | 4 (5.9%) | 2 (1.5%) | — | — |
| Clinical staging |  |  |  |  |
| Clinical T stage | 2 [1–3] | 2 [1–3] | 0.958 | 0.024 |
| Node-positive (cN+)^a^ | 30 (44.1%) | 44 (33.6%) | 0.165 | 0.217 |
| Year of surgery | 2021 [2020–2022] | 2021 [2020–2023] | 0.496 | 0.095 |

Data are median [interquartile range] or n (%). SMD = standardized mean difference; values < 0.10 indicate adequate balance.

a SMD = 0.217; nodal status imbalance reflects the limited availability of node-positive controls in the non-neoadjuvant NSCLC registry cohort and is acknowledged as a limitation.
